# Understanding Molecular Landscape of Endometrial Cancer through Next Generation Sequencing: What We Have Learned so Far?

**DOI:** 10.3389/fphar.2016.00409

**Published:** 2016-11-01

**Authors:** Siti-Syazani Suhaimi, Nurul-Syakima Ab Mutalib, Rahman Jamal

**Affiliations:** UKM Medical Molecular Biology Institute, Universiti Kebangsaan MalaysiaCheras, Malaysia

**Keywords:** next generation sequencing, endometrial cancer, molecular landscape, precision medicine, screening, early diagnosis

## Abstract

Endometrial cancer (EC) is among the most common gynecological cancers affecting women worldwide. Despite the early detection and rather high overall survival rate, around 20% of the cases recur with poor prognosis. The Next Generation Sequencing (NGS) technology, also known as massively parallel sequencing, symbolizes a high-throughput, fast, sensitive and accurate way to study the molecular landscape of a cancer and this has indeed revolutionized endometrial cancer research. Understanding the potential, advantages, and limitations of NGS will be crucial for the healthcare providers and scientists in providing the genome-driven care in this era of precision medicine and pharmacogenomics. This mini review aimed to compile and critically summarize the recent findings contributed by NGS technology pertaining to EC. Importantly, we also discussed the potential of this technology for fundamental discovery research, individualized therapy, screening of at-risk individual and early diagnosis.

## Overview of Endometrial Cancer

Endometrial cancer (EC) ranks as the sixth most frequent cancers among women worldwide with around 320, 000 reported cases and 76, 000 deaths (International Agency for Research on Cancer [Iarc], 2014). This cancer is normally detected early with a relatively high overall survival rate (Sgo Clinical Practice Endometrial Cancer Working Group et al., 2014). However, nearly one fifth of the cases have poor prognosis with a median survival of about 1 year (Obel et al., 2006; Salvesen et al., 2012). Unopposed estrogen therapy, estrogen producing tumors, tamoxifen, obesity, nulliparity, diabetes mellitus, and early onset of menstruation are among the risk factors associated with EC (Brinton et al., 2005).

Bokhman (1983) was the first to propose the pathogenetic dualistic model of two different types of endometrial carcinoma, named as type I and type II. Type I endometrioid endometrial cancer (EEC) is driven by estrogen and represents most of sporadic cases (Bansal et al., 2009). EEC typically occurs in premenopausal and younger postmenopausal women who often diagnosed with low-grade well-differentiated tumor thus carrying a better prognosis (Soliman et al., 2005; Garg and Soslow, 2014). On the contrary, the type II non-endometrioid endometrial carcinoma (NEEC) accounts for only 10–20% of sporadic endometrial carcinoma with no underlying estrogen exposure (Doll et al., 2008). NEEC is commonly diagnosed in older postmenopausal women, who typically present with advanced-stage disease and poor prognosis (Amant et al., 2005). It is also associated with high mortality and reduced survival rates (Hamilton et al., 2006; Mendivil et al., 2009). This classification is imperfect as the minority of EC characteristic of both groups may overlap because of heterogeneity of this disease, especially the high grade EEC (Murali et al., 2014). Characterization of molecular landscapes will give insights into tumor classification, which may influence treatment recommendations and provides prospects for precision medicine. Therefore, there is a need to explore the molecular landscape of EC treatment using the next generation sequencing (NGS) approaches.

Lynch Syndrome (LS) is a hereditary cancer syndrome caused by germline alterations in the DNA mismatch repair (MMR) genes (Tafe et al., 2014). Those with LS will have an increased risk of colon cancer. Despite being overlooked in association with LS, individuals with this syndrome also has 20–60% risk of developing EC (Tafe et al., 2014). The mutation frequencies of MMR genes are: 50–66% in *MSH2*, 24–40% in *MLH1*, 10–13% in *MSH6* and <5% in *PMS2* (Wang et al., 2013).

## Overview of Next Generation Sequencing (NGS) Technology

The emergence of NGS three decades after Sanger sequencing represents the potential to dramatically revolutionize biomedical research by enabling the high throughput comprehensive analysis of genomes and transcriptomes at an inexpensive scale (Shendure and Ji, 2008). Compared with Sanger sequencing, NGS technologies offer extraordinarily high throughput capacity which reduces cost per base, time and has enabled the discovery of both common and rare variants with a much deeper sequencing read coverage (Koboldt et al., 2013). NGS is also a versatile technology which enables various applications including whole genome sequencing (WGS) for model and non-model organisms (Ng and Kirkness, 2010), whole exome sequencing (Rabbani et al., 2014), targeted resequencing (Meldrum et al., 2011) as well as analysis of coding and non-coding RNA expression, alternative splicing and discovery of novel non-coding RNAs (Wang et al., 2009). In addition, while 30× coverage for WGS is considered as the standard (Rehm et al., 2013), low-pass WGS with coverage less than 10× had also been used to assess structural variation (Cancer Genome Atlas Research Network et al., 2013).

**FIGURE 1:**
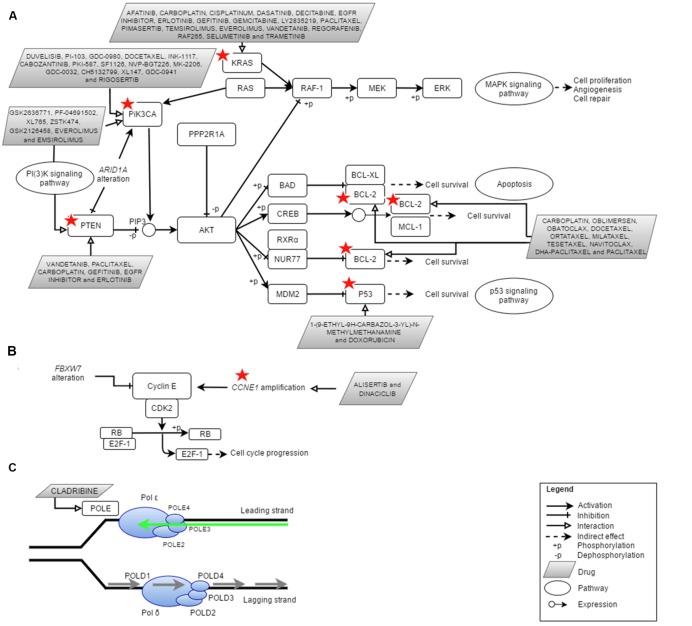
**Cellular pathways currently being targeted in the treatment of endometrial cancer (EC) and the potential actionable genes in EC identified via Next Generation Sequencing (NGS).** Red star depicted genes with clinically cancer-relevant drug interactions as defined by DGIdb (Wagner et al., 2016). **(A)** PI(3)K, MAPK, p53 and apoptosis pathway involving *PIK3CA*, *PTEN*, *PPP2R1A*, *KRAS*, *P53*, and *BCL2*, **(B)** cell cycle pathway involving *FBXW7* and *CCNE1* as well as **(C)** DNA replication pathway in eukaryote which involves *POLE* (DNA polymerase 𝜀). Figures were adapted from Kanehisa et al. (2016) and Seshagiri (2013). Adapted by permission from Macmillan Publishers Ltd: [NATURE GENETICS] (Seshagiri), copyright (2013).

Over the past 4 years, NGS technologies were applied in EC research (**Table 1**). NGS has promoted and improved the detection of key types of molecular alterations such as single nucleotide substitutions, small insertions, and deletions, copy number alterations, structural variations and novel transcripts. New drug targets were identified thus providing the oncologists with various potential options in treating EC patients (**Figure 1**). Here, we provide an overview of the use of NGS for subtype classification, identification of potential diagnostic biomarkers and screening of therapeutic targets for personalized treatment of EC.

**Table 1:** Main findings contributed by Next Generation Sequencing (NGS) in endometrial cancers (ECs).

Method	Samples	Main findings	Study
Low-pass whole genome sequencing	ECs and matched DNA from normal tissues or blood (*n* = 106 pairs)	Recurrent translocations of genes in WNT, EGFR–RAS–MAPK, PI(3)K, protein kinase A, retinoblastoma and apoptosis pathway. The most frequent translocations in the member of BCL family (BCL2, BCL7A, BCL9, and BCL2L11).	Cancer Genome Atlas Research Network et al., 2013
Whole exome sequencing	ECs and matched DNA from normal tissues or blood (*n* = 248 pairs)	Frequent mutations in *PTEN*, *CTNNB1*, *PIK3CA*, *ARID1A*, *KRAS* and novel mutations in *ARID5B*. Significant increase of transversion mutation frequency and novel hotspot mutations in *POLE* in a subset of endometrioid cancers.	
Whole exome sequencing	EC and matched DNA from blood (*n* = 13 pairs)	Mutation on *ARID1A* are associated with PI3K pathway activation.	Liang et al., 2012
Whole exome sequencing	Uterine serous cancer and matched normal tissues (*n* = 13 pairs)	Mutation on chromatin-remodeling and ubiquitin ligase complex genes.	Le Gallo et al., 2012
Whole exome sequencing	Uterine serous cancer and matched DNA from blood or tissue samples (*n* = 10 pairs)	Mutation on *FBXW7* and amplification of *CCNE1* locus (encodes cyclin E, substrate of FBXW7).	Kuhn et al., 2012
Whole exome sequencing	Uterine serous cancer (*n* = 52) and matched DNA from blood (*n* = 34)	Mutation on *SPOP*, *CDH4*, *TAF1*, amplification of *CCNE1* and loss of *MBD3*.	Zhao et al., 2013
Targeted gene sequencing (nine genes)	Low-grade EEC (*n* = 276), grade 3 EEC (*n* = 30), serous (*n* = 37) and carcinosarcoma subtype (*n* = 42)	Distinct mutation frequency on *PTEN* and *TP53* on low-grade EEC and grade 3 EEC. Significantly different mutations frequency on *PTEN*, *ARID1A*, *PPP2R1A*, *TP53*, and *CTNNB1* between grade 3 EEC and serous carcinoma.	McConechy et al., 2012
Targeted gene sequencing (seven genes)	EEC (*n* = 307) and ovarian endometrioid cancer (*n* = 33)	Distinct mutation profile in *PTEN* and *CTNNB1*.	McConechy et al., 2014
Targeted gene sequencing (578 genes)	EC (*n* = 10)	Frequent mutations in *PTEN* (50%) and genes involved in the endometrial cancer-related molecular pathway including IL-7 signaling pathway.	Chang et al., 2016
RNA sequencing	ECs (*n* = 333)	Three clusters; mitotic, hormonal and immunoreactive	Cancer Genome Atlas Research Network et al., 2013
Small RNA sequencing	ECs (*n* = 367)	Six miRNA clusters significantly associated with *MLH1* hypermethylation (miR-148a and miR-375), histology, grade (miR-21) and stage.	
RNA sequencing	Stage I EEC and adjacent normal tissues (*n* = 3 pairs)	First report on dysregulation of miRNAs (hsa-miR-196a-5p, hsa-miR-328-3p, hsa-miR-337-3p, and hsa-miR-99a-3p) in EC.	Xiong et al., 2014
Small RNA sequencing	Normal, hyperplastic, and EC biopsies (*n* = 10 trios)	Definition of sncRNAs signature (1229 miRNAs, 10 piRNAs and three SnoRNAs) involved in neoplastic transformation.	Ravo et al., 2015
Paired end RNA sequencing	EC with matched non-cancerous tissue (*n* = 9 pairs)	Significant upregulation of fusion gene TSNAX-DISC1 in EC which formed through splicing without chromosomal rearrangement.	Li et al., 2014

*EEC, Endometrial endometrioid carcinoma; sncRNAs, Small non-coding RNAs; miRNAs, MicroRNAs; piRNAs, Piwi-interacting RNA; SnoRNAs, Small nucleolar RNAs*.

Whole genome sequencing provides a comprehensive view of the cancer genome including all types of somatic/germline mutations, nucleotide substitutions, small insertions and deletions, copy number variations, chromosomal rearrangements, as well as analysis of the non-coding regions (Meyerson et al., 2010). Since WGS is still expensive, laborious and produces a massive amount of data for analysis and interpretation, whole exome sequencing (WES) which concentrates only on the protein-coding exon of human genome is a preferred alternative (Rabbani et al., 2014).

However, despite being high throughput, most of the information obtained from WGS and WES are functionally unclear and the genetic alterations could be just possible passenger mutations with unknown clinical significance (Meldrum et al., 2011). Besides, WGS and WES are unsuitable for clinical application because of relatively lower read depth and this later limit the identification of low-allelic-fraction single nucleotide polymorphisms (SNPs) which is important for early diagnosis, prevention of drug resistance and residual tumor detection (Xu et al., 2014). Therefore, deep sequencing of targeted gene provides better alternative in clinical setting due to several advantages including higher coverage for increased analytical sensitivity and specificity, generate feasible and interpretable data, significant cost reduction, and shorter turnaround time (Xu et al., 2014). Targeted gene sequencing also has proven useful for a large number of applications including studying disease-relevant gene subsets, diagnostic testing for hereditary disorders and therapeutic decision-making for somatic cancers (Rehm, 2013).

Since last decade, EC transcriptomes have been dominantly investigated using hybridization-based microarray techniques (Maxwell et al., 2005; Cohn et al., 2010; Ratner et al., 2010). The advent of NGS has also revolutionized cancer transcriptomics studies. RNA and miRNA sequencing have rapidly emerged as powerful tools for genome wide expression profiling and offer several advantages over conventional microarray.

## Whole Genome and Whole Exome Sequencing

To date, there is only one publication in EC by The Cancer Genome Atlas (TCGA) group which utilized WGS to characterize the chromosomal aberration in 106 ECs (Cancer Genome Atlas Research Network et al., 2013). Recurrent translocations of genes in important cancer pathways including WNT, EGFR–RAS–MAPK, PI3K, protein kinase A, retinoblastoma and apoptosis were identified (Cancer Genome Atlas Research Network et al., 2013). The most frequent translocations were discovered in a member of the BCL family which were *BCL2*, *BCL7A*, *BCL9*, and *BCL2L11* (Cancer Genome Atlas Research Network et al., 2013). This large-scale consortium also performedWES on 248 ECs and identified novel *POLE* hotspot mutations (Pro286Arg and Val411Leu) in 13 of the 17 ultra-mutated samples (Cancer Genome Atlas Research Network et al., 2013). In addition, *PTEN*, *PIK3R1*, *PIK3CA*, *FBXW7*, and *KRAS* were found to be significantly mutated. This was the largest genomic characterization of EC so far and their findings propose a reclassification that might assist in personalized treatment for patients with aggressive phenotype.

So far there are four published EC studies utilizing WES (**Table 1**). Liang et al. (2012) was the first to perform WES in 13 ECs with matched normal DNA. In combination with functional genomics, the authors identified 12 potential driver genes including 10 tumor suppressor genes (ARID1A, INHBA, KMO, TTLL5, GRM8, IGFBP3, AKTIP, PHKA2, TRPS1, and WNT11) and two oncogenes (ERBB3 and RPS6KC1). Concentrating on *ARID1A*, mutation profiles were integrated with functional proteomics in additional 222 EC samples, demonstrating the role of *ARID1A* as a novel regulator of PI3K pathway activity (Liang et al., 2012).

While Liang et al. (2012) characterized the exomes of ECs without stratification of grade and histological subtypes, another group (Le Gallo et al., 2012) focused on the rare but aggressive serous carcinoma subtype of EC which has high recurrence rate, poor survival and more likely to present with metastatic disease due to resistance to chemotherapy (Hamilton et al., 2006). Le Gallo et al. (2012) performed WES on 13 matched pairs of serous ECs and normal tissues. In addition to confirming the existence of alterations in *TP53*, *PIK3CA* and *PPP2R1A*, they also identified high frequency of somatic alterations in novel genes involved in serous ECs which were FBXW7, CHD4, SPOP, MAP3K4, ABCC9, and CYP4X1 (Le Gallo et al., 2012). Chromatin remodeling and ubiquitin ligase complex pathways were found to be the most perturbed pathways which have been associated with frequent alterations in set of 11 chromatin remodeling genes including CHD4 gene and component of ubiquitin ligase complex (FBXW7 and SPOP gene), respectively (Le Gallo et al., 2012).

In the same year, Kuhn et al. (2012) performed WES on 10 uterine serous carcinomas with matched normal blood or tissue samples. In addition to confirming the previous discovery by Le Gallo et al. (2012), they also identified high mutation frequencies of *FBXW7* and genes known to be associated with serous EC including TP53, PIK3CA, and PPP2R1A. DNA copy number analysis revealed concurrent frequent genomic amplification of the *CCNE1* with *FBXW7* mutations, suggesting that these genes are involved in same signaling pathway (Kuhn et al., 2012). They also proposed the role of endometrial intraepithelial carcinoma as a precursor to serous carcinoma whereby nine cases of serous carcinoma with an associated serous endometrial intraepithelial carcinoma had concordant *PIK3CA*, *PP2R1A*, and *TP53* mutations (Kuhn et al., 2012). Taken together, the authors presented molecular genetic alterations involving the p53, cyclin E-FBXW7 and PI3K pathways as the major mechanisms in the uterine serous carcinoma progression.

The WES findings from both rather underpowered studies by Kuhn et al. (2012) and Le Gallo et al. (2012) were later supported by Zhao et al. (2013) using five times more samples. Fifty two uterine serous cancer patients of which 34 had matched normal tissue samples were subjected to WES (Zhao et al., 2013). In addition to earlier published studies which identified frequent alterations in *TP53*, *PIK3CA*, *PPPR1A*, and *FBXW7*, Zhao et al. (2013) also discovered high frequency mutations of the SPOP gene which targets the protein for ubiquitination. Interestingly, this study also reported alterations in TF1 gene, an element of the core TFIID transcriptional machinery which may result in overexpression of cyclin D and subsequently promote cell cycle progression and proliferation (Zhao et al., 2013). Frequent *TP53* deletions and amplifications of chromosome segments containing *PIK3CA* and *CCNE1* are known to be targets of FBXW7 (Kuhn et al., 2012). This study also discovered overexpression of *ERBB2* which act as an upstream regulator of PIK3CA/AKT/mTOR signaling pathway and loss of MBD3 gene function, a member of the NuRD-chromatin-modification complex (Zhao et al., 2013). To summarize, all of these studies which employed WGS and WES reported specific pathways that are frequently mutated in ECs including DNA damage, chromatin remodeling, cell cycle, and cell proliferation pathways.

## Targeted Gene Sequencing

Two different studies by McConechy et al. (2012) took on to improve the controversial classification among pathological subtypes endometrial carcinoma (endometrioid, serous, carcinosarcoma, mixed, and clear cell) and between histological endometrioid types of EC and ovarian cancer using a panel of genes related to both cancers. The first study involved targeted enrichment sequencing on a large cohort of 393 EC samples using a nine-gene panel: ARID1A, PPP2R1A, PTEN, PIK3CA, KRAS, CTNNB1, TP53, BRAF and PPP2R5C (McConechy et al., 2012). Both low-grade and grade 3 endometrioid EC demonstrated a similar pattern of high frequency of mutations in *PTEN*, *PIK3CA*, *ARID1A*, *KRAS*, and *CTNNB1* (McConechy et al., 2012). There is a significant increase in *TP53* mutation frequency in grade 3 endometrioid EC when compared to low-grade EC (McConechy et al., 2012). *ARID1A* mutation was significantly associated with concurrent *PTEN* and *PIK3CA* mutations in both low-grade endometrioid EC and grade 3 endometrioid ECs, suggesting a cooperative role in endometrioid carcinogenesis (McConechy et al., 2012). However, this phenomenon was not observed in endometrial serous carcinomas which have frequent mutations in *TP53* and *PPP2R1A* but lack mutations in *PTEN*, *ARID1A*, and *CTNBB1*. On the other hand, in carcinosarcoma, two distinct mutation profiles have been identified which were the endometrioid type (*PTEN*, *PIK3CA*, *ARID1A*, and *KRAS* mutations) and serous type (*TP53* and *PPP2R1A*; McConechy et al., 2012).

Using a panel of seven well-characterized genes in ECs (*ARID1A, PTEN, PIK3CA, KRAS, CTNNB1, PPP2R1A*, and *TP53)*, McConechy’s research group compared two morphologically similar cancer types, endometrial endometrioid carcinoma (*n* = 307) and ovarian endometrioid carcinoma (*n* = 33) using exon capture sequencing (McConechy et al., 2014). Higher frequency of mutations was discovered in low-grade endometrial endometrioid carcinoma (67%) compared to low-grade ovarian endometrioid carcinoma (17%; McConechy et al., 2014). The frequency of *CTNNB1* mutation was significantly higher in ovarian endometrioid cancer (53%) when compared to endometrioid EC (28%). However, mutations frequencies in other genes were not significantly different between both cancers. They hypothesized that the different mutation spectrum in both cancers might be because of different exposures to the microenvironment during carcinogenesis, whereby the ovarian endometrioid carcinoma thrives in a highly oxidative environment that promotes tumorigenesis (Kobayashi et al., 2009). Consistent with other previously discussed studies, distinct mutation patterns in PI3K and WNT signaling pathways were identified, suggesting therapeutic opportunities for treatment of EC (McConechy et al., 2014).

The most recently published endometrial research using NGS involved 10 Taiwanese EC patients (Chang et al., 2016). The exomes of 578 cancer-related genes were captured and deep-sequenced using NGS, resulting in average of 500× coverage. This study revealed 120 variants in 99 genes, with 50% of the ECs harboring mutations in the *PTEN* (Chang et al., 2016). Molecular aberrations in *PIK3R1*, *AKT2* and *FOXO1* that could led to putative activation of the IL-7 signaling pathway and which has not been previously linked with EC, are also reported.

Targeted NGS represents a potential to be adopted in clinical laboratory practices and have diagnostic applications for screening of individual at risk for developing EC (Tafe, 2015). To date, there are seven NGS-based assays that include LS-associated genes offered by several reference laboratories including Ambry Genetics (ColoNext; 14 genes), ARUP (Gastrointestinal Hereditary Cancer Panel; 15 genes), Baylor Miraca Genetics Laboratories (High Risk Hereditary Colorectal Cancer Panel; 12 genes), GeneDx (OncoGeneDx: Lynch/Colorectal Cancer High Risk Panel; seven genes), Mayo Medical laboratories (Hereditary Colon Cancer Multi-Gene Panel; 17 genes), Myriad (myRisk^TM^ Hereditary Cancer Panel; 25 genes) and University of Washington (ColoSeq^TM^; 23 genes; Tafe, 2015). However, the important challenges of NGS include interpreting incidental findings, genetic counseling, and getting informed consent from the patients. These calls for development of clinical practice guidelines for test development, validation, reporting, and reporting of incidental findings (Tafe, 2015).

## RNA and miRNA Sequencing

Via RNA sequencing and unsupervised k-means clustering of 333 ECs, Cancer Genome Atlas Research Network et al. (2013) identified three robust clusters which were mitotic, hormonal and immunoreactive subtypes. The mitotic subtype was characterized by *TP53* alteration and mostly comprised of serous/mixed histology and endometrioid grade 3 while both of hormonal and immunoreactive subtypes were predominantly comprised of endometrioid grade 1/2 and *PTEN* mutated patients (Cancer Genome Atlas Research Network et al., 2013). Interestingly, the hormonal subtype exhibited upregulation of hormone related genes (ESR1, PGR and downstream targets) which could make the patients more responsive to hormonal therapy (Cancer Genome Atlas Research Network et al., 2013), implying potential for individualized treatment. In addition, the group performed unsupervised consensus clustering of miRNA expression profiles in 367 ECs and discovered six clusters which some of them were significantly associated with *MLH1* hypermethylation (miR-148a and miR-375), histology, grade (miR-21) and stage.

Xiong et al. (2014) simultaneously characterized the transcriptome of both mRNAs and miRNAs using RNA sequencing on three pairs of stage 1 endometrioid EC and adjacent non-cancerous tissue (Xiong et al., 2014). By integrating expression data of mRNAs and miRNAs, they identified a total of 438 target pairs which were inversely correlated including 320 dysregulated genes. Downstream pathway enrichment analysis revealed six differently expressed miRNAs (hsa-let-7c-5p, hsa-miR-196a-5p, hsa-miR-328-3p, hsa-miR-337-3p, and hsa-miR-99a-3p, hsa-miR-181c-3p) targeting 11 differently expressed genes (E2F5, CDKN2A, CCNA2, TP53, BUB1B, CCNE1, CDK1, MCM4, SKP2, CDC6 and TGFB3*)* in the cell cycle pathway (Xiong et al., 2014).

A genome wide characterization of small non-coding RNAs (sncRNAs) in EC carcinogenesis was performed on biopsies of normal (*n* = 10), hyperplastic (*n* = 6) and tumor Type 1 endometrial tissues (*n* = 10) using RNA sequencing (Ravo et al., 2015). Significant patterns in sncRNA expression between the sample groups were identified and led to the discovery of sncRNAs signature (129 miRNAs, 10 piRNAs, and three snoRNAs) which is said to be involved in neoplastic transformation (Ravo et al., 2015). Integrated gene expression profiling with the aberrantly expressed sncRNAs signature revealed their involvement in multiple signaling pathway including ERK/MAPK, TGF-β and Wnt/β-catenin in both hyperplastic and neoplastic tissues (Ravo et al., 2015).

Gene fusion refers to an aberrant rearrangement between two genes which encode a new fusion protein, serving as a strong driver mutation in cancer (Mertens et al., 2015). Through pair-end RNA sequencing in nine pairs of EC and matched non-cancerous tissues, a fusion gene called chimeric translin-associated X-disrupted-in-schizophrenia (TSNAX-DISC1) in EC was discovered (Li et al., 2014). This fusion transcript was formed by intragenic splicing and its expression was further validated in 176 paired ECs and matched non-cancerous tissues. Dysregulation of *TSNAX* is presumed to be associated with cancer and the authors proposed the potential of *TSNAX–DISC1* as an EC biomarker (Li et al., 2014).

## Conclusion

In this mini review, we compiled and concisely review the literatures using NGS in basic EC research. NGS has indeed revolutionized EC genomics by enabling discovery of the major alterations in the genome which could serve as potential biomarkers for prognosis and drug development. The discovery of these biomarkers by NGS has the potential to accelerate application of genome-guided information into precision medicine and pharmacogenomics. Despite limited publications so far, NGS assays are already actively adopted for routine clinical testing in molecular pathology laboratories for identification of individual at risk of developing EC.

## Author Contributions

S-SS and N-SA drafted this manuscript. N-SA and RJ were responsible for idea conception, critical evaluation and manuscript review.

## Conflict of Interest Statement

The authors declare that the research was conducted in the absence of any commercial or financial relationships that could be construed as a potential conflict of interest.
